# Poster Session I - A183 DIAGNOSTIC AND THERAPEUTIC UTILITY OF LIVER BIOPSY IN CHILDREN: A SINGLE-CENTER RETROSPECTIVE STUDY

**DOI:** 10.1093/jcag/gwaf042.183

**Published:** 2026-02-13

**Authors:** E Altamimi, D Almomani, J Harb, I Al-Kawalit, F Alkhalili

**Affiliations:** Pediatrics and Neonatology, Jordan University of Science and Technology Faculty of Medicine, Irbid, Irbid Governorate, Jordan; Jordan University of Science and Technology Faculty of Medicine, Irbid, Irbid Governorate, Jordan; Jordan University of Science and Technology Faculty of Medicine, Irbid, Irbid Governorate, Jordan; Jordan University of Science and Technology Faculty of Medicine, Irbid, Irbid Governorate, Jordan; Pediatrics and Neonatology, Jordan University of Science and Technology Faculty of Medicine, Irbid, Irbid Governorate, Jordan

## Abstract

**Background:**

Despite advances in non-invasive diagnostics, liver biopsy (LB) remains central to pediatric Hepatology. Its current diagnostic yield and therapeutic impact warrant re-evaluation in contemporary practice.

**Aims:**

To evaluate the diagnostic yield, concordance with pre-biopsy clinical impressions, and influence on the management of liver biopsies in children.

**Methods:**

A retrospective review was conducted of all patients aged 0–18 years who underwent LB at King Abdullah University Hospital between January 2017 and March 2024. Demographic, clinical, procedural, histopathologic, and outcome data were extracted. Biopsies were categorized by intent (diagnostic, intraoperative, or other) and outcome (confirmed diagnosis, new/alternate diagnosis, normal histology, or inconclusive). A management change was defined as the initiation, cessation, or major alteration of therapy prompted by biopsy results. Descriptive statistics summarized the data.

**Results:**

Eighty-three patients were included (median age 1.4 years; mean 5.8 years), most aged <6 months. Cholestasis was the leading indication (36.1%). Biopsies were diagnostic in 71.1%, intraoperative in 25.3%, and other in 3.6%; the percutaneous approach predominated (74.7%). Histopathology yielded a definitive diagnosis in 75.9% (confirming the pre-biopsy impression in 50.6% and revealing a new or alternate diagnosis in 25.3%). Normal histology was seen in 1.2%, and 19.3% were inconclusive. Biopsy findings led to management changes in 50.6% of cases. 22.9% complication rate was recorded. Major bleeding occurred in 10.8%, PICU admission in 4.82%, and two deaths (2.4%) followed the procedure.

**Conclusions:**

Liver biopsy in children provided a high diagnostic yield and influenced management in most cases, underscoring its enduring clinical value despite the growth of non-invasive modalities. The notable complication rate highlights the importance of careful patient selection and procedural optimization to maximize benefits and minimize risks

**Funding Agencies:**

NoneNone

